# Exploring the Intersection of Gender Identity and Eating Behaviours in Gender‐Diverse Populations

**DOI:** 10.1002/erv.70055

**Published:** 2025-11-08

**Authors:** Paolo Meneguzzo, Michela Biscaro, Laura Vozzi, Marina Bonato, Alberto Scala, Marina Miscioscia, Andrea Garolla, Angela Favaro, Gender Incongruence Interdisciplinary Group (GIIG)

**Affiliations:** ^1^ Department of Neuroscience University of Padova Padova Italy; ^2^ Padova Neuroscience Center University of Padova Padova Italy; ^3^ Regional Reference Center for Gender Incongruence Azienda Ospedale‐Università di Padova Padova Italy; ^4^ Department of Developmental Psychology and Socialization University of Padova Padova Italy; ^5^ Department of Medicine Unit of Andrology and Reproductive Medicine University of Padova Padova Italy

**Keywords:** cognitive restraint, eating behaviours, emotional eating, gender expression, gender identity, minority stress, non‐binary, transgender

## Abstract

**Background:**

Gender‐diverse individuals are at elevated risk for disordered eating, shaped by body dissatisfaction, gender dysphoria, and minority stress. The roles of gender identity and expression, however, remain underexplored in clinical samples. This study examined how self‐perceived masculinity, femininity, and gender variance relate to eating behaviours in gender‐diverse adults seeking gender‐affirming care.

**Methods:**

Eighty‐four adults assigned female (*n* = 50) or male (*n* = 34) at birth were recruited from a regional gender incongruence clinic. Participants self‐identified as male (*n* = 44), female (*n* = 30), or non‐binary (*n* = 10); 36 were receiving gender‐affirming hormone therapy (GAHT). They completed the Three‐Factor Eating Questionnaire (TFEQ) and the Gender Variance Scale (GVS), assessing masculinity, femininity, and deviation from cisnormative expectations. Regression models tested associations between gender traits and eating behaviours.

**Results:**

Masculinity was positively associated with cognitive restraint, while femininity correlated with emotional and uncontrolled eating. Gender variance was not significantly related to TFEQ; however, exploratory analyses suggested a modest quadratic trend with dysregulated eating. Eating behaviours did not differ by GAHT status.

**Conclusions:**

Gender expression, more than gender variance, appears to influence eating behaviours in gender‐diverse individuals. Clinicians should consider how masculinity‐ and femininity‐linked eating patterns shape assessment and intervention.

## Summary

- Self‐perceived masculinity was associated with greater cognitive restraint, while femininity predicted higher emotional and uncontrolled eating in gender‐diverse adults.
- Gender variance showed a nonlinear (quadratic) relationship with dysregulated eating, suggesting elevated risk at intermediate levels of variance.
- Gender‐affirming hormone therapy status did not significantly influence eating behaviour patterns, underscoring the role of psychosocial gender expression factors.

## 1 Introduction

Transgender and gender‐diverse (TGD) individuals—those whose gender identity or expression differs from sex assigned at birth—include transgender men, transgender women, and non‐binary people, representing a spectrum of gendered experiences (Hammack and Wignall 2023).

Research consistently shows that TGD individuals face an elevated risk of disordered eating and eating disorders (EDs), with prevalence rates exceeding those observed in cisgender populations (Diemer et al. 2018; Ferrucci et al. 2022). Several pathways may account for this vulnerability, including gender dysphoria, body dissatisfaction, and minority stress resulting from stigma and discrimination (Hartman‐Munick et al. 2021; Meneguzzo, Zuccaretti, et al. 2024; Mezza et al. 2024). Eating behaviours can also emerge as strategies to align the body more closely with gender identity or with socially enforced ideals—for example, reducing feminine fat distribution in transmasculine individuals or minimizing muscularity in transfeminine individuals (Rasmussen et al. 2023).

Gender‐affirming medical interventions may alleviate some of these risks by reducing body uneasiness and enhancing congruence between gender identity and physical appearance (Cahill et al. 2025; Doyle et al. 2023; Loria et al. 2024). Access to hormone therapy has been associated with improvements in body image and, in some cases, reductions in disordered eating (Fisher et al. 2014; Rasmussen et al. 2025; Testa et al. 2017). However, findings remain mixed: longitudinal studies in adolescents report no significant short‐term changes in eating pathology after initiating care (Pham et al. 2023), and large‐scale cohort data suggest that hormone therapy may decrease eating disorder risk in transmasculine individuals but increase it in transfeminine individuals (Loria et al. 2024). Moreover, recent evidence highlights that disordered eating behaviours are prevalent even among those seeking gender‐affirming treatment and often persist despite transition‐related interventions (Usta Sağlam et al. 2024). This complexity underscores that medical transition alone cannot fully explain the persistence and variability of eating difficulties in TGD populations. Beyond bodily modification, psychosocial processes such as minority stress, stigma, and the internalization of transphobic attitudes exert a powerful influence on how individuals experience and regulate their bodies (Castellini et al. 2023; Meneguzzo, Vozzi, et al. 2024a). Recognising this interplay highlights the need to situate disordered eating not only within the domain of medical affirmation, but also within broader cultural and psychological contexts that shape vulnerability and resilience.

Much of the existing literature continues to conceptualize gender as a categorical identity, contrasting TGD and cisgender individuals. Such an approach, while informative, overlooks within‐group differences. Dimensional perspectives—considering self‐perceived masculinity, femininity, and gender variance (the extent to which one diverges from cisnormative expectations)—offer a richer framework to understand how gendered embodiment intersects with cultural body ideals and eating behaviours (Heiden‐Rootes et al. 2023; Yarrow et al. 2024). Yet empirical studies using validated dimensional measures remain scarce.

The present exploratory study addresses this gap by examining associations between self‐perceived masculinity, femininity, gender variance, and specific eating behaviours—cognitive restraint, uncontrolled eating, and emotional eating—in a clinical sample of TGD adults. By shifting from categorical to dimensional measures, the study seeks to refine understanding of eating behaviours in this population and to inform gender‐sensitive approaches to assessment and intervention.

## 2 Methods

### 2.1 Participants

This study enrolled 84 adults (≥ 18 years) attending the Regional Reference Center for Gender Incongruence [MASKED] between September and December 2024. The Center provides multidisciplinary psychological, endocrinological, and social support for individuals seeking medical gender affirmation [citation MASKED]. All participants self‐identified as transgender men, transgender women, or non‐binary and received a DSM‐5‐TR diagnosis of gender dysphoria, defined as a marked incongruence between experienced gender and sex assigned at birth, accompanied by clinically significant distress.

Inclusion criteria were: (1) age ≥ 18 years; (2) self‐identification as transgender or gender‐diverse; and (3) adequate Italian language proficiency. Exclusion criteria were: (1) inability to provide informed consent; and (2) acute psychiatric conditions that could compromise participation.

The study focused exclusively on TGD individuals to investigate within‐group variability, given that their embodied experiences of gender and social perception differ qualitatively from cisgender populations. Participants represented heterogeneous stages of gender affirmation, with and without ongoing gender‐affirming hormone therapy (GAHT).

All participants gave written informed consent. The protocol was approved by the [MASKED] Ethics Committee and conducted in accordance with the Declaration of Helsinki.

### 2.2 Instruments

Demographic and anthropometric data included age, height, weight, and current use of GAHT. Body Mass Index (BMI) was calculated from self‐reported height and weight and considered as a potential covariate in analyses.

To assess eating behaviours, participants completed the Three‐Factor Eating Questionnaire—Revised 18‐item version (TFEQ‐R18) (De Lauzon et al. 2004; Rossi et al. 2024). This self‐report scale evaluates three dimensions: cognitive restraint (intentional restriction to control weight/shape), uncontrolled eating (loss of control in response to cues), and emotional eating (eating triggered by negative affect). The TFEQ‐R18 is not a diagnostic tool for eating disorders but rather a dimensional measure of eating patterns. It is widely used in both clinical and non‐clinical populations and is particularly suited for exploratory research in transgender and gender‐diverse individuals, where full‐threshold eating disorders may be underdiagnosed or present atypically.

Gender identity–related traits were assessed with the Gender Variance Scale (GVS) (Yarrow et al. 2024; Meneguzzo et al. 2025). The GVS consists of 10 items assessing self‐perceived masculinity and femininity across four domains (feel, look, do, interests) on a 9‐point Likert scale. Separate masculinity and femininity scores are obtained (range: 5–45), an overall gender variance score (range: 0–80) that reflects the degree to which one's self‐perception diverges from the gendered expectations associated with sex assigned at birth. While higher scores generally indicate greater variance relative to binary gender norms, nonbinary individuals often report lower totals. This is not because they are closer to cisnormativity, but because their experiences do not align neatly with either binary pole, which the scale was originally designed to capture. The Italian validation has confirmed its good psychometric properties, supporting its use as a dimensional, person‐centred tool to investigate gender expression and variance in clinical populations.

All questionnaires were completed in digital format during clinical visits and required approximately 15–20 min.

### 2.3 Statistical Analysis

Descriptive statistics (means, standard deviations, frequencies) were computed for all variables. Group comparisons by gender identity and GAHT status were tested with independent‐samples *t*‐tests and one‐way ANOVAs, with Bonferroni corrections for post hoc tests; chi‐square tests were used for categorical variables.

Pearson correlations examined associations among TFEQ subscales, GVS scores (masculinity, femininity, gender variance), BMI, age, and GAHT duration. A MANOVA tested the effect of GAHT status (receiving vs. not receiving) on eating behaviour scores.

To evaluate predictors of eating behaviours, three multiple linear regression models were run with cognitive restraint, emotional eating, and uncontrolled eating as dependent variables. Masculinity, femininity, and gender variance were entered as predictors, controlling for age, BMI, and GAHT status. Model assumptions were met (normality, linearity, homoscedasticity, absence of multicollinearity, VIF < 2).

Potential nonlinear relationships between gender variance and TFEQ subscales were tested with curve estimation, comparing linear and quadratic models.

Analyses were conducted in SPSS (v25.0); significance was set at *p* < 0.05 (two‐tailed).

## 3 Results

### 3.1 Participant Characteristics and Group Comparisons

Participants were 84 individuals with an average age of 25.90 ± 8.11 years and an average BMI of 23.72 ± 5.36. The sex assigned at birth was female for 50 participants and male for 34. Self‐recognized gender identities included 44 identifying as male, 30 as female, and 10 as non‐binary. Of this sample, 36 participants were undergoing gender‐affirming hormonal therapy (GAHT+), from 1 month to 120 months, while 48 were not (GATH−).

No significant differences were found when comparing participants based on GAHT status, except for the GVS, where higher scores were observed in individuals in the GATH + group. See Table 1 for details.

**TABLE 1:** Demographic and psychometric characteristics of participants by GAHT status.

	GATH− *n* = 48	GATH+ *n* = 36	t *p*
Age, years	24.81	27.36	1.434
	(7.90)	(8.27)	0.155
BMI, kg/m^2^	23.39	24.15	0.638
	(5.84)	(4.69)	0.525
Gender			
Male	24	20	2.432^a^
Female	16	14	0.296
Nonbinary	8	2	
Cognitive restrain	13.54	11.78	1.789
	(5.14)	(3.90)	0.077
Uncontrolled eating	19.27	19.72	0.300
	(6.84)	(6.82)	0.765
Emotional eating	7.46	6.64	1.430
	(2.70)	(2.45)	0.157
Masculine scale	27.08	26.83	0.095
	(11.47)	(12.57)	0.925
Femminine scale	20.33	21.61	0.504
	(10.54)	(12.65)	0.615
GVS	58.29	63.89	2.281
	(13.38)	(9.08)	0.025

*Note:* Values are reported as means (standard deviations). *t*‐tests were used for continuous variables; gender distribution was tested with chi‐square (χ^2^).

Abbreviations: BMI = Body Mass Index; GAHT = Gender‐affirming hormone therapy; GVS = Gender Variance Scale.

^a^ chi‐square.

### 3.2 Group Differences in Eating Behaviours by Gender Traits

Using gender as a comparative factor, we found significant differences in the TFEQ subscales. Significant associations were found: masculinity scores were positively associated with cognitive restraint, and femininity scores were positively associated with emotional and uncontrolled eating. See Table 2 for details.

**TABLE 2:** Demographic and psychometric characteristics by gender identity.

	Male *n* = 44	Female *n* = 30	Nonbinary *n* = 10	F *p*	Post hoc
Age, years	24.16	28.20	26.70	2.340	
	(5.27)	(10.68)	(8.59)	0.103	
BMI, kg/m^2^	24.75	22.63	22.42	1.766	
	(6.51)	(3.62)	(2.95)	0.177	
Cognitive restrain	13.75	11.07	13.70	3.289	M > F (0.046)
	(5.45)	(3.11)	(3.97)	0.042	
Uncontrolled eating	19.41	21.27	14.30	4.256	F > N (0.014)
	(6.30)	(7.62)	(2.91)	0.017	
Emotional eating	6.59	8.30	2.54	5.832	F > M (0.014)
	(2.54)	(2.56)	(5.80)	0.004	F > N (0.021)
Masculine scale	36.32	13.87	25.20	136.085	M > F (< 0.001)
	(6.02)	(4.62)	(7.52)	< 0.001	M > N (< 0.001)
					N > F (< 0.001)
Feminine scale	12.00	33.87	21.00	148.291	F > M (< 0.001)
	(5.20)	(5.71)	(4.94)	< 0.001	F > N (< 0.001)
					N > M (< 0.001)
GVS	64.32	60.73	44.60	14.618	M > N (< 0.001)
	(10.66)	(9.84)	(10.97)	< 0.001	F > N (< 0.001)

*Note:* Values are reported as means (standard deviations). Between‐group differences were analysed using one‐way ANOVA with Bonferroni post hoc comparisons.

Abbreviations: BMI = Body Mass Index; F = female; M = male; N = non‐binary.

### 3.3 Effects of GAHT Duration and Correlation Patterns

The results of the MANOVA indicated that the duration of GAHT did not have a statistically significant effect on emotional eating (*F* = 1.652, *p* = 0.092). However, significant effects were observed for uncontrolled eating (*F* = 2.428, *p* = 0.009) and emotional eating (*F* = 1.952, *p* = 0.038).

**FIGURE 1:**
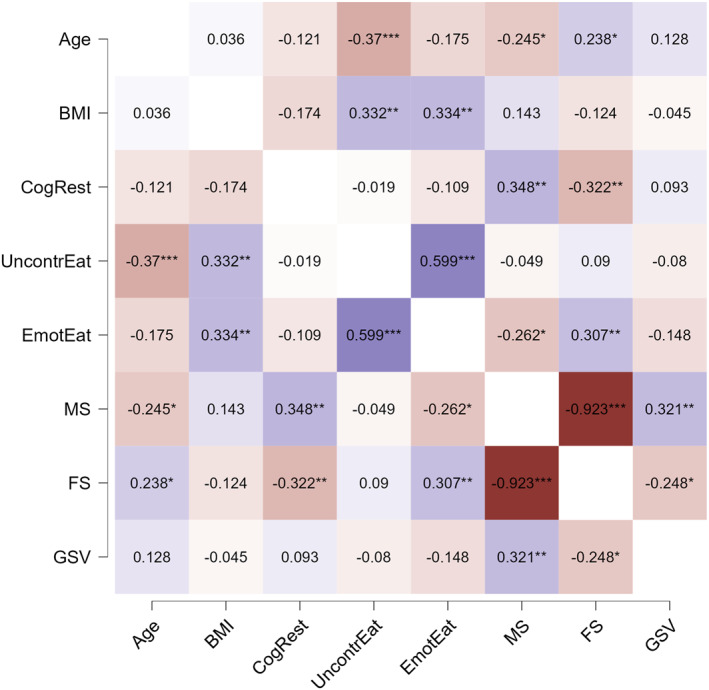
Correlation matrix for the TFEQ subscales: Cognitive Restraint (CogRest), Uncontrolled Eating (UncontrEat), and Emotional Eating (EmotEat), as well as BMI, age, and gender‐related traits: Masculine Subscale (MS), Feminine Subscale (FS), and Gender Variance Scale (GVS). Purple indicates positive correlations, while orange represents negative correlations. The intensity of the colour reflects the strength of the relationship, with darker shades representing stronger correlations. Significant correlations are highlighted, showing the associations between eating behaviors, gender identity, and body‐related variables.

Figure 1 shows the correlation matrix between the TFEQ subscales (uncontrolled eating, emotional eating, cognitive restraint), BMI, age, and GSV. Significant correlations were observed between TFEQ subscales, GVS scores, BMI, and age. Masculinity correlated positively with cognitive restraint; femininity correlated positively with emotional and uncontrolled eating. Age correlated negatively with uncontrolled eating and masculinity, and positively with femininity. BMI correlated positively with uncontrolled and emotional eating. The duration of GAHT was not significantly associated with any of the eating behaviour dimensions and is therefore not included in the figure for clarity.

### 3.4 Regression Analyses Predicting Eating Behaviours

Three multiple linear regression analyses were conducted with cognitive restraint, emotional eating, and uncontrolled eating as dependent variables. Predictors were masculinity, femininity, and gender variance, controlling for age, BMI, and GAHT status.

For cognitive restraint, the model was significant, F (6, 77) = 3.22, *p* = 0.007, adjusted *R* ^2^ = 0.14. Masculinity (*β* = 0.347, *p* = 0.004) and BMI (*β* = 0.296, *p* = 0.018) were significant predictors; other variables were nonsignificant.

For emotional eating, the model was significant, F (6, 77) = 6.76, *p* < 0.001, adjusted *R* ^2^ = 0.29. Femininity (*β* = 0.314, *p* = 0.017) and BMI (*β* = 0.313, *p* = 0.014) were significant; other predictors were nonsignificant.

For uncontrolled eating, the model was significant, F (6, 77) = 5.95, *p* < 0.001, adjusted *R* ^2^ = 0.26. Femininity (*β* = 0.276, *p* = 0.042) and BMI (*β* = 0.405, *p* = 0.001) were significant; other predictors were nonsignificant.

Gender variance was not a significant predictor in any model. See Table 3 for details.

**TABLE 3:** Regression analysis of predictors for TFEQ subscales.

Predictor	Cognitive restraint β (*p*)	Emotional eating β (*p*)	Uncontrolled eating β (*p*)
Masculinity subscale	0.35 (0.004)	−0.01 (0.951)	−0.03 (0.850)
Femininity subscale	−0.16 (0.191)	0.31 (0.017)	0.28 (0.042)
GVS	−0.03 (0.807)	0.12 (0.380)	0.07 (0.655)
Age	0.16 (0.209)	0.01 (0.938)	−0.07 (0.592)
BMI	0.30 (0.018)	0.31 (0.014)	0.41 (0.001)
GAHT status	−0.07 (0.552)	−0.05 (0.699)	−0.07 (0.590)
*F* (df)	3.22 (6, 77)	6.76 (6, 77)	5.95 (6, 77)
Adjusted *R* ^*2*^	0.14	0.29	0.26

Abbreviations: *β* = standardized regression coefficien; tGAHT = Gender‐affirming hormone therapy.

### 3.5 Curvilinear Associations Between Gender Variance and Eating Behaviours

Curve estimation analyses were conducted with gender variance as the independent variable and TFEQ subscales as dependent variables.

For cognitive restraint, no significant associations were found (linear: F (1,82) = 0.72, *p* = 0.399; quadratic: F (2,81) = 0.71, *p* = 0.493).

For emotional eating, the quadratic model was significant, F (2,81) = 5.81, *p* = 0.004, adjusted *R* ^2^ = 0.104; both the linear (*β* = 0.557, *p* = 0.005) and quadratic terms (*β* = −0.005, *p* = 0.003) were significant.

For uncontrolled eating, the quadratic model was also significant, F (2,81) = 5.81, *p* = 0.004, adjusted *R* ^2^ = 0.104, with significant linear (*β* = 0.557, *p* = 0.005) and quadratic (*β* = −0.005, *p* = 0.003) components.

## 4 Discussion

This study examined the associations between gender‐related traits and eating behaviours in a gender‐diverse sample, adopting a dimensional approach. By combining the TFEQ with the GVS, we explored how self‐perceived masculinity, femininity, and gender variance may relate to cognitive restraint, emotional eating, and uncontrolled eating. The results should be interpreted as exploratory, but they provide preliminary indications that gender expression—particularly masculinity and femininity—may be more closely related to eating behaviours than gender variance or GAHT duration.

Stronger masculine identification was associated with higher cognitive restraint and lower emotional eating. These patterns are consistent with previous research linking masculinity to ideals of discipline and control (DeGue et al. 2024; Mahalik et al. 2003). While such associations have been mostly described in cisgender populations, our findings suggest that they may also apply to transgender and gender‐diverse individuals, who navigate similar sociocultural frameworks (Anzani et al. 2024). Conversely, femininity was positively associated with emotional and uncontrolled eating and negatively associated with restraint. These results resonate with studies showing that femininity is often linked with greater body dissatisfaction, emotion‐focused coping, and vulnerability to disordered eating (Grabe et al. 2008; Tiggemann 2004).

Gender variance did not emerge as a predictor in linear models, but exploratory curve estimation revealed nonlinear associations with both emotional and uncontrolled eating. The quadratic pattern suggested greater difficulties at intermediate levels of variance, indicating that individuals whose gender expression diverges moderately from binary norms may be particularly vulnerable. This position may reflect a phase of identity ambiguity or intensified exposure to societal pressures enforcing binary categories, both of which can foster distress and dysregulated eating behaviours (McArthur et al. 2025; Mezza et al. 2024). By contrast, individuals with low scores—often nonbinary participants in our sample—showed lower levels of emotional and uncontrolled eating. This finding does not necessarily imply lower distress, but rather suggests that their eating experiences may manifest through other pathways not fully captured by these specific eating behaviour dimensions. At higher levels of variance, protective factors such as community support, identity consolidation, or more resilient coping may buffer against dysregulated eating (Hammack and Wignall 2023; Wittlin et al. 2025). Although exploratory, these findings highlight the value of dimensional approaches in capturing heterogeneity within gender‐diverse populations and suggest that different forms of disordered eating may be differentially linked to gender variance.

Neither GAHT status nor duration were associated with eating behaviours. This differs from some previous studies reporting improvements in body image and eating concerns after GAHT (Castellini et al. 2023; Nobili et al. 2018). One possible explanation is that our focus on eating behaviours across a spectrum, rather than on the presence of a specific eating disorder diagnosis, captures patterns that may be more directly shaped by internalized gender norms, coping strategies, and minority stress than by medical transition alone. This perspective aligns with evidence showing that psychosocial determinants, including internalized transphobia, continue to influence disordered eating even in the context of gender‐affirming care (McArthur et al. 2025; Urban et al. 2024). Finally, age was negatively associated with uncontrolled eating, in line with research suggesting that emotion regulation and body image concerns tend to improve with maturity (Yeomans et al. 2024).

Taken together, these exploratory findings suggest that gender expression may contribute to eating behaviour patterns in ways that are not fully explained by identity categories or medical transition.

### 4.1 Clinical Implications

Although preliminary, these findings may have some implications for clinical practice. Assessment of eating disorders in gender‐diverse individuals may benefit from considering how patients position themselves in relation to cultural expectations of masculinity and femininity, rather than focusing only on weight and shape concerns. For example, restraint may be influenced by ideals of control associated with masculinity, while emotional or uncontrolled eating may reflect expectations tied to femininity. Interventions might therefore include a focus on internalized gender norms, encouraging reflection on how such norms shape coping strategies and body experiences. The nonlinear association with gender variance also suggests that risk may not increase uniformly across levels of variance, highlighting the need for individualized assessment. The absence of significant effects of GAHT points to the importance of psychological support alongside medical transition, with attention to identity integration and coping mechanisms.

### 4.2 Limitations

The cross‐sectional design prevents causal inference, and the modest sample size may have limited detection of subtle effects. A post hoc sensitivity check confirmed adequate power only for medium‐to‐large effects, so null findings should be interpreted cautiously. Self‐report measures may also introduce bias and fail to capture lived experiences. Finally, although the Italian GVS provides a validated dimensional tool, it does not encompass stigma, identity conflict, or embodiment distress that may shape eating behaviours.

## 5 Conclusion

In conclusion, this exploratory study suggests that gender expression, particularly masculinity and femininity, may influence eating behaviours among gender‐diverse individuals, while gender variance showed a more complex, nonlinear association that warrants further examination. By adopting a dimensional perspective, our findings may also help to contextualize the mixed results reported on changes in eating pathology following gender‐affirming interventions, highlighting that medical transition alone may not capture the full range of factors shaping eating behaviours. These results should be regarded as preliminary and hypothesis‐generating, given the study's limitations, but they point toward the value of integrating dimensional measures of gender into future research. Larger, longitudinal studies are needed to clarify these mechanisms and to inform more nuanced, gender‐sensitive clinical approaches.

## Author Contributions

P.M., M.M., A.G. and A.F. designed the study and wrote the protocol. P.M., M.B., L.V., A.S. and M.B. collected data. P.M. undertook the statistical analysis. P.M. wrote the first draft of the manuscript. All authors contributed to the revision and have approved the final manuscript.

## Funding

The authors have nothing to report.

## Ethics Statement

All individuals included in this study provided informed consent. The study protocol received approval from the local Ethics Committee and complies with the ethical principles outlined in the Declaration of Helsinki and its subsequent amendments.

## Conflicts of Interest

The authors declare no conflicts of interest.

## Data Availability

The datasets used and/or analysed during the current study are available from the corresponding author on reasonable request.
